# Diagnostic ability of mid-upper arm circumference-to-length ratio in detecting wasting among infants aged 1–6 months in Ethiopia

**DOI:** 10.1017/jns.2022.21

**Published:** 2022-03-21

**Authors:** Beshada Rago Jima, Hamid Yimem Hassen, Paluku Bahwere, Seifu Hagos Gebreyesus

**Affiliations:** 1 Department of Nutrition and Dietetics, School of Public Health, Addis Ababa University, Addis Ababa, Ethiopia; 2 Department of Primary and Interdisciplinary Care, Faculty of Medicine and Health Sciences, University of Antwerp, Antwerp, Belgium; 3 Centre of Research in Epidemiology, Biostatistics and Clinical Research, School of Public Health, Free University of Brussels, 808, Route de Lennik, 1070 Brussels, Belgium

**Keywords:** Diagnosis, Infant, MUAC-to-length, ROC and wasting, AUC, area under ROC curve, IQR, interquartile range, MUAC, mid-upper arm circumference, MUAC/L, mid-upper arm circumference-to-length, ROC, receiver operating characteristic, WHO, World Health Organization, WLZ, weight-for-length *Z-*score

## Abstract

Mid-upper arm circumference (MUAC) is an age-sensitive anthropometric measurement in infants. However, exact age is difficult to know, particularly in low-income countries. We evaluated the diagnostic accuracy of an age-independent mid-upper arm circumference-to-length (MUAC/L) ratio measurement in detecting wasting among infants aged 1–6 months in Ethiopia. A facility-based diagnostic accuracy study was conducted on 467 in-patient infants aged 1–6 months from March to May 2019. The receiver operating characteristic (ROC) curve was used to evaluate the ability of MUAC/L to detect wasting. Sensitivity, specificity, positive likelihood ratio, negative likelihood ratio and positive and negative predictive values were calculated. The magnitude of severe wasting was 21⋅6 % and moderate wasting was 13⋅0 %. The area under the ROC curve (AUC) of MUAC/L was 0⋅77 (95 % CI 0⋅73, 0⋅81) for detecting moderate wasting and 0⋅92 (95 % CI 0⋅89, 0⋅94) for detecting severe wasting. MUAC/L had a sensitivity of 91⋅1 % (95 % CI 81⋅3, 94⋅4), a specificity of 84⋅7 % (95 % CI 80⋅6, 88⋅2), a positive likelihood ratio of 5⋅82 (95 % CI 4⋅53, 7⋅48) and a negative likelihood ratio of 0⋅13 (95 % CI 0⋅07, 0⋅22) in total infants. The optimal MUAC/L cut-off was <0⋅190 for boys and <0⋅185 for girls. MUAC/L had an AUC of 0⋅77 and 0⋅92 in predicting moderate and severe wasting in infants aged 1–6 months, respectively. Using MUAC/L to treat Ethiopian infants with severe wasting and infants with similar characteristics in other countries could improve treatment coverage.

## Introduction

Wasting in infants under the age of 6 months is becoming more widely recognized, and it is associated with higher mortality rates as compared with older children^(1)^. An estimated 8⋅5 million infants under the age of 6 months are wasted in low- and middle-income countries (LMICs), with 3⋅8 million severely wasted^(2)^. Inadequate breastfeeding, low birth-weight, infections, children born in remote areas and newborns born to malnourished mothers are all common risk factors for under-nutrition in infants under 6 months old^(3–5)^. Infant malnutrition can increase vulnerability to sickness, create chronic non-communicable diseases and impair future physical, social and mental development^(6,7)^. As a result, prompt detection and referral are critical in LMICs such as Ethiopia, where wasting is a serious public health issue.

The World Health Organization (WHO) recommends utilizing weight-for-height/length (WFH/L), the appearance of bilateral pitting oedema and measuring mid-upper arm circumference (MUAC) to identify acute malnutrition in children aged 6–59 months^(8)^. The diagnosis of wasting among infants aged under 6 months is currently based on the weight-for-length *Z*-score (WLZ) using the same threshold applied in older children^(9)^. MUAC has recently been identified as a wasting indication in infants under the age of 6 months^(10–12)^. However, because of the rapid growth experienced throughout childhood, MUAC has become age- and sex-dependent anthropometric assessments^(13)^. Our prior research also reveals that the age of the infant influences MUAC's diagnostic efficacy in detecting infants with severe wasting^(12)^. In recognition of this age dependency, several authors have devised and recommended the use of a MUAC-for-age indication^(14,15)^. However, in many circumstances, determining the individuals' ages is problematic, prompting numerous attempts to develop anthropometric data interpretation methods that do not require knowledge of age or at least accurate age.

The mid-upper arm circumference-to-length (MUAC/L) ratio is among the anthropometric indicators not requiring age knowledge. The method is justified by the fact that nutritionally labile tissues of the upper arm are considerably reduced by hunger, whereas length and skeletal measures are more genetically controlled and are much less influenced by malnutrition^(16–19)^. Hence, the present study aimed to evaluate the diagnostic accuracy of the mid-upper arm-to-length ratio in identifying infants with wasting.

## Materials and methods

### Study design and site

We conducted a facility-based cross-sectional study in in-patient units of four public hospitals (Hiwot Fana Specialized and Teaching Hospital, Haramaya General Hospital, Bisdimo Hospital and Garamuleta General Hospital) in Eastern Ethiopia from March to May 2019. Eastern Ethiopia is among areas prone to drought, conflict and food insecurity, which have been among the key determinants of under-nutrition in the country for several decades^(20)^. The protocol for the management of acute malnutrition used in all selected hospitals is in line with the recommended WHO management of acute malnutrition guidelines^(9)^.

### Study participants

Infants aged 1–6 months admitted to four selected public hospitals in Easter Ethiopia were eligible for the study. Infants with a recumbent length of <45 cm were excluded from the study as WLZ could not be calculated. Infants who showed clinical evidence of bilateral pitting oedema during the study period were also excluded as the measurement of weight in the presence of oedema is misleading.

### Sample size and sampling procedure

The diagnostic accuracy test formula was used to calculate the required sample size^(21)^.
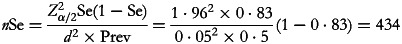

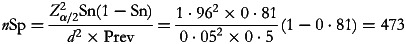


where Se is the sensitivity, Sp is the specificity, *d* is the marginal error Prev is the prevalence with assumptions of a sensitivity of 83 %, a specificity of 81 %^(22)^, a magnitude of 50 % (as there was no published estimate of wasting among the study age groups in the setting) and using a precision of 5 % and a non-response rate of 10 %. The largest sample size was determined to be 521 infants.

A total of four public hospitals with large client flow and stabilization center were deemed necessary to get an adequate sample size. A multi-stage sampling technique was employed to select the sample. Using probability proportional to sample size, the required sample size was allocated for selected hospitals. After the required sample size was allocated to each hospital, eligible infants were selected using a systematic random sampling (every second subject is admitted to in-patient setting) until the required sample size was obtained.

### Data collection

Proficient nurses performed anthropometric measurements (weight, length and MUAC) in line with the WHO standard anthropometric techniques^(23)^. Each measurement was completed twice by the same measurer and the mean of the readings was recorded. The discrepancies of the two readings greater than the tolerable difference (>0⋅5 cm for MUAC, >0⋅5 kg for weight and >1 cm for length) were resolved by obtaining the measurements for the third time.

Age was calculated by subtracting the date of birth from the date of data collection. The infant's date of birth was obtained from the mother's report. In the difficulty of taking the infant's birth date from the mother's report, a local event calendar and/or patient immunization card were used. To minimize incorporation bias and to keep the reliability of measurements, infants' clinical and previous anthropometric information were not available for data collectors.

### MUAC measurement

To measure MUAC, we used non-stretch infant MUAC tape (UNICEF Ross Laboratories, Columbus, OH, USA) manufactured for clinical diagnosis purposes. The infant's left arm was bent to 90° at the elbow, and the midpoint between the tip of the shoulder and the tip of the elbow was identified and marked. The MUAC strip was then placed comfortably around the marked midpoint of the relaxed arm, and the measurement was recorded to the nearest 0⋅1 cm.

### Weight measurement

To measure weight, we used a digital scale (UNICEF scale S0141015 by SECA Ltd, Birmingham, UK) that has a precision of 100g, positioning the scale with all its feet touching the flat ground. We used the training function of the scale, in which the infant's mother/caretaker stepped on the scale without the infant, the scale was zeroed, and then the infant was handed to the mother/caretaker. Weight measurements were recorded to the nearest 0⋅1 kg.

### Length measurement

To measure length, we used the portable wooden length board (Shorr Productions, Woonsocket, RI, USA) and placed on the hard and flat surface. Infants were measured with the head, back, buttocks and heels touching the surface of board; heels together; knees extended and head in the Frankfort horizontal plane. Measurements were taken to the nearest 0⋅1 cm.

To define wasting (moderate and severe), we used the WHO growth standard^(24)^. Infants with WLZ below −3 sd from the median were classified as severely wasted, whereas infants with WLZ below −2 but no lower than −3 sd were classified as moderately wasted. MUAC was divided by length to obtain a ‘unitless’ MUAC/L ratio.

Eight professional nurses received refresher anthropometric measurement training for 5 d and went through standardization exercises before the assessments began. The data collectors obtained the repeated measurements of ten infants. The reliability of the measurements was evaluated by calculating inter- and intra-observer technical error of measurements (TEM). The inter-observer technical errors of measurement for weight (kg) is 0⋅132, length (cm) is 0⋅374 and MUAC (cm) 0⋅001. Additionally, the coefficient of reliability was calculated, and we found 94⋅9 % for weight, 98⋅9 % for length and 98⋅9 % for MUAC. Compared with reference values, all TEM were within the acceptable range^(25)^.

### Data analysis

Data were double entered into EpiData version 1.2.2.0 (EpiData Association) to detect any errors or omissions that might have occurred. The validated and cleaned EpiData database was converted into STATA format. Analyses were conducted using STATA version 15.1 (StataCorp LP). The WLZ was computed using a WHO Anthro version 3.2.2 software based on sex and age according to the WHO-2006 growth standard and later on appended to the STATA file. The normality of continuous data (age, weight, length, MUAC, WLZ and MUAC/L) was checked using the Shapiro–Wilk test and the *Q*–*Q* plot. The data were deviated from normality (*P* < 0⋅001), and Mann–Whitney *U-*test was performed to compare the median age, weight, length, MUAC and WLZ between boys and girls. Categorical variables were summarized using frequency and percentage, whereas numerical variables were reported using median with interquartile range (IQR). The Mann–Whitney *U-*test was done to compare the median age, weight, length, MUAC, WLZ and MUAC/L between boys and girls. The Spearman's rank correlation coefficient was used to measure the strength of association between MUAC/L and age, weight, length, MUAC and WLZ. Statistical significance was declared at *P* < 0⋅05.

The receiver operating characteristic (ROC) curves were constructed to evaluate the predictive performance of MUAC/L for moderate and severe wasting in terms of area under the curve (AUC) with a 95 % confidence interval (CI). The level of accuracy was determined using AUC as: 0⋅50–0⋅60 bad, 0⋅60–0⋅70 sufficient, 0⋅70–0⋅80 good, 0⋅80–0⋅90 very good and 0⋅90–1⋅0 excellent performance^(26)^. To examine the variation in the accuracy of MUAC/L across sex, sub-group analysis was performed for boys and girls separately. The sensitivity, specificity, positive likelihood ratio, negative likelihood ratio, positive predictive value and negative predictive values with 95 % CIs at different MUAC/L cut-off were calculated. The Youden's Index *J* (sensitivity + specificity − 1) identified the optimum cut-off to classify the infants as moderately and severely wasted.

## Results

Of the 521 hospitalized infants eligible for consideration in the study, seventeen infants were excluded for the following reasons: caretakers thought infants were severely ill (*n* 11); caretakers thought infants were very young (*n* 6). Of the total 504 (96⋅7 %) enrolled infants, 37 (6⋅9 %) had WLZ data flagged out of the plausible bound (less than −5) and excluded from the whole analysis. Overall, the data of 241 (51⋅6 %) male and 226 (48⋅4 %) female infants were included in the analysis. The median age was 3⋅3 (IQR: 2⋅3–4⋅8) months for all included infants. The median WLZ and MUAC/L were −0⋅12 (IQR: −2⋅66 to 0⋅34) and 0⋅20 (IQR: 0⋅19 to 0⋅21), respectively.

Above one-fifth, 101 (21⋅6 %) and 61 (13⋅0 %) infants were severely and moderately wasted, respectively. The magnitude of severe wasting was not significantly different between boys and girls (19⋅9  and 23⋅4 %, respectively; *P =* 0⋅424) (Table 1).

**Table 1.** Median comparison of age, weight, length, MUAC, WLZ and MUAC/L between boys and girls and their nutritional status

Characteristics	Male (*n* 241)	Female (*n* 226)	*P*-value^a^	Total (*n* 467)
	Median (IQR)	Median (IQR)		Median (IQR)
Age (months)	3⋅2 (2⋅2–4⋅7)	3⋅3 (2⋅3–4⋅8)	0⋅515	3⋅3 (2⋅3–4⋅8)
Weight (kg)	5⋅4 (4⋅5–6⋅0)	5⋅0 (4⋅0–5⋅9)	0⋅059	5⋅2 (4⋅2–6⋅0)
Length (cm)	58⋅5 (55⋅0–63⋅0)	58⋅2 (54⋅5–62⋅0)	0⋅399	58⋅4 (55–62⋅5)
MUAC (mm)	120 (107–130)	116 (100–130)	0⋅115	119 (103–130)
WLZ	−1⋅08 (−2⋅57–0.39)	−1⋅30 (−2⋅8–0⋅19)	0⋅325	−1⋅27 (−2⋅6–0⋅34)
MUAC/L	0⋅203 (0⋅181–0⋅216)	0⋅199 (0⋅214–0⋅174)	0⋅233	0⋅200 (0⋅216–0⋅159)
Nutritional status, *n* (%)				
Wasting	79 (32⋅7)	83 (36⋅6)	0⋅371	162 (34⋅6)
Severe	48 (19⋅9)	53 (23⋅4)	0⋅424	101 (21⋅6)
Moderate	31 (12⋅8)	30 (13⋅2)	0⋅898	61 (13⋅0)
Normal	162 (67⋅2)	143 (63⋅2)	0⋅278	305 (65⋅4)

IQR, interquartile range; MUAC, mid-upper arm circumference; MUAC/L, mid-upper arm circumference-to-length ratio; WLZ, weight-for-length *Z*-score.

a *P*-values are based on Mann–Whitney *U-*test.

### Relationship between MUAC/L, WLZ, weight, length and age

The MUAC/L showed a positive correlation with WLZ, *rs* 0⋅71 (95 % CI 0⋅66, 0⋅75) and weight, *rs* 0⋅55 (95 % CI 0⋅48, 0⋅61), but not with age, *rs* 0⋅001 (95 % CI −0⋅09 to 0⋅09). The correlation between MUAC/Land age was much weaker than the correlation between MUAC and age, *rs* 0⋅23 (95 % CI 0⋅14, 0⋅31). The correlation between length and age was *rs* 0⋅44 (95 % CI 0⋅36, 0⋅51).

### Diagnostic accuracy of MUAC/L to identify moderate and severe wasting

**Fig. 1.**
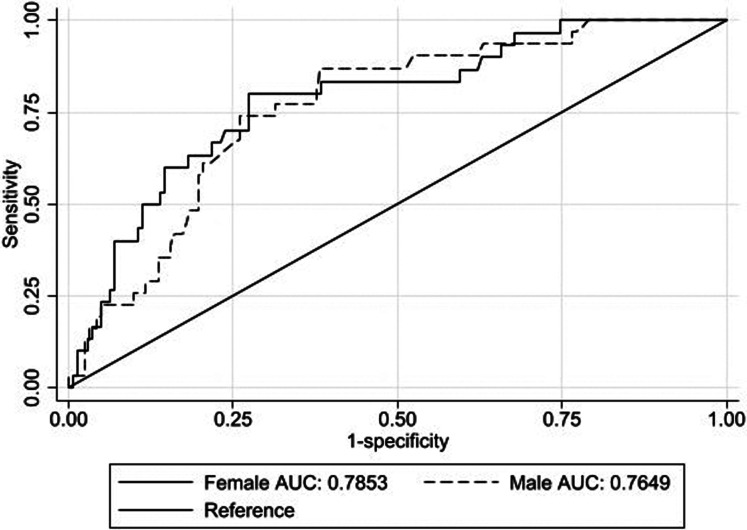
Receiver's operating characteristic curve of mid-upper arm circumference-to-length (MUAC/L) ratio for detecting moderate wasting against weight-for-length in boys and girls.

**Fig. 2.**
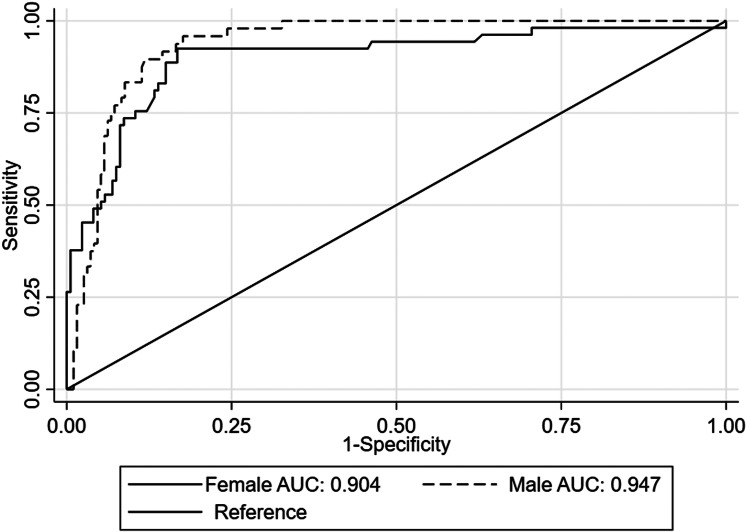
Receiver's operating characteristic curves of mid-upper arm circumference-to-length (MUAC/L) ratio for detecting severe wasting against weight-for-length in boys and girls.

The AUC of MUAC/L to identify moderate and severe wasting per sex is presented. The AUC of MUAC/L to identify moderately wasted infants was 0⋅76 (95 % CI 0⋅70, 0⋅82) in boys and 0⋅78 (95 % CI 0⋅72, 0⋅84) in girls (Fig. 1). Whereas, to detect severely wasted infants, the AUC was 0⋅94 (95 % CI 0⋅90, 0⋅96) in boys and 0⋅90 (95 % CI 0⋅85, 0⋅93) in girls (Fig. 2). Overall, MUAC/L has an AUC of 0⋅77 (95 % CI 0⋅73, 0⋅81) for detecting moderate wasting and 0⋅92 (95 % CI 0⋅89, 0⋅94) for detecting severe wasting.

The accuracy of different cut-offs of MUAC/L to identify moderate and severe wasting is shown in Table 2. Across the lower range of MUAC/L cut-offs (0⋅175–0⋅185), specificity was high (93⋅8–88⋅5 % for moderate and 90⋅4–84⋅7 % for severe wasting), but sensitivity was low (24⋅6–34⋅4 % for moderate and 76⋅2–91⋅1 % for severe wasting); with higher cut-offs (0⋅210–0⋅220), sensitivity increased substantially (85⋅2–96⋅7 % for moderate and 97⋅0–99⋅0 % for severe wasting) but at the expense of specificity (24⋅6–49⋅5 % for moderate and 21⋅0–43⋅7 % for severe wasting).

**Table 2.** Diagnostic accuracy measures for different cut-offs of MUAC/L ratio for detecting moderate and severe wasting among infants aged 1–6 months (*n* 467)

MUAC/L	Moderate wasting			Severe wasting		
Cut-offs	Sensitivity	Specificity	YI	Sensitivity	Specificity	YI
<0⋅220	96⋅7	24⋅6	0⋅213	99⋅0	21⋅0	0⋅200
<0⋅215	90⋅2	36⋅4	0⋅266	98⋅0	32⋅0	0⋅300
<0⋅210	85⋅2	49⋅5	0⋅347	97⋅0	43⋅7	0⋅407
<0⋅205	80⋅3	60⋅3	0⋅406	97⋅0	53⋅6	0⋅506
<0⋅200	73⋅8	69⋅8	0⋅436	95⋅0	62⋅6	0⋅576
<0⋅195	54⋅1	80⋅7	0⋅348	94⋅1	74⋅9	0⋅690
<0⋅190	42⋅6	85⋅9	0⋅285	93⋅1	80⋅9	0⋅740
<0⋅185	34⋅4	88⋅5	0⋅229	91⋅1	84⋅7	0⋅758
<0⋅180	31⋅1	92⋅5	0⋅236	83⋅2	88⋅8	0⋅720
<0⋅175	24⋅6	93⋅8	0⋅189	76⋅2	90⋅4	0⋅666

MUAC/L, mid-upper arm circumference; YI, Youden index.

Table 3 summarizes the diagnostic test accuracies for optimal MUAC/L cut-offs to detect moderate and severe wasting. Using the Youden index, the optimal cut-off MUAC/L to detect moderate wasting was <0⋅206 for boys and <0⋅198 for girls, and the optimal cut-off for severe wasting was <0⋅190 for boys and <0⋅185 for girls. Overall, the MUAC/L cut-off <0⋅185 provided high sensitivity and specificity in predicting severe wasting (sensitivity of 91⋅1 % (95 % CI 81⋅3, 94⋅4 %) and specificity of 84⋅7 % (95 % CI 80⋅6, 88⋅2 %)) in total included infants.

**Table 3.** Optimal cut-off values, the area under the ROC curves, sensitivities, specificities, positive and negative likelihood ratio and positive and negative predictive values of MUAC/L associated with severe wasting among infants aged 1–6 months

Sex	Nutritional status	*n* (%)	Cut-off level	AUC (95 % CI)	Sensitivity (95 % CI)	Specificity (95 % CI)	LR+ (95 % CI)	LR− (95 % CI)	PPV (95 % CI)	NPV (95 % CI)
Boys	Moderate wasting	31 (12⋅8)	<0⋅206	0⋅70 (0⋅61, 0⋅78)	77⋅4 (58⋅9, 90⋅4)	62⋅3 (54⋅4, 69⋅8)	2⋅06 (1⋅56, 2⋅71)	0⋅36 (0⋅19, 0⋅70)	30⋅1 (24⋅6, 36⋅1)	93⋅0 (87⋅2, 96⋅2)
	Severe wasting	48 (19⋅9)	<0⋅190	0⋅88 (0⋅84, 0⋅93)	93⋅8 (82⋅8, 98⋅7)	82⋅9 (76⋅8, 87⋅9)	5⋅48 (3⋅98, 7⋅54	0⋅07 (0⋅02, 0⋅23)	57⋅7 (49⋅7, 65⋅2)	98⋅2 (94⋅7, 99⋅4)
Girls	Moderate wasting	30 (13⋅3)	<0⋅198	0⋅72 (0⋅63, 0⋅81)	70⋅0 (50⋅6, 85⋅3)	74⋅1 (66⋅1, 81⋅1)	2⋅71 (1⋅88, 3⋅89)	0⋅40 (0⋅23, 0⋅70)	34⋅2 (26⋅5, 42⋅7)	92⋅8 (88⋅1, 95⋅7)
	Severe wasting	53 (23⋅4)	<0⋅185	0⋅86 (0⋅81, 0⋅91)	88⋅7 (77⋅0, 95⋅7)	83⋅2 (76⋅8, 88⋅5)	5⋅29 (3⋅74, 7⋅47)	0⋅14 (0⋅06, 0⋅29)	61⋅8 (53⋅4, 69⋅5)	96⋅0 (91⋅9, 98⋅1)
Total	Moderate wasting	61 (13⋅0)	<0⋅201	0⋅72 (0⋅66, 0⋅78)	73⋅8 (60⋅9, 84⋅2)	69⋅8 (64⋅3, 74⋅9)	2⋅45 (1⋅95, 3⋅07)	0⋅38 (0⋅24, 0⋅58)	32⋅9 (28⋅1, 38⋅1)	93⋅0 (89⋅7, 95⋅3)
	Severe wasting	101 (21⋅6)	<0⋅185	0⋅87 (0⋅83, 0⋅90)	91⋅1 (81⋅3, 94⋅4)	84⋅7 (80⋅6, 88⋅2)	5⋅82 (4⋅53, 7⋅48)	0⋅13 (0⋅07, 0⋅22)	61⋅6 (55⋅5, 67⋅3	96⋅6 (94⋅2, 98⋅0)

AUC, area under ROC curve; CI, confidence interval; LR+, positive likelihood ratio; LR−, negative likelihood ratio; MUAC/L, mid-upper arm circumference; NPV, negative predictive value; PPV, positive predictive value; ROC, receiver operating characteristic.

The magnitude of severe wasting using both WLZ < −3 and MUAC/L < 0⋅185 was 85⋅1 %. This character is different across sex: the optimal MUAC/L cut-off <0⋅190 identified 81⋅2 % of severely wasted boys and the optimal MUAC/L cut-off <0⋅185 identified 88⋅7 % of severely wasted girls. The magnitude of moderate wasting using both WLZ and MUAC/L was 75⋅9 % in the whole infants.

## Discussion

The present study evaluated the diagnostic performance of MUAC/L to detect wasting (moderate and severe wasting) among hospitalized infants aged 1–6 months. The MUAC/L was strongly correlated with WLZ for both boys and girls. The observed AUC 0⋅92 (95 % CI 0⋅89, 0⋅94) shows that MUAC/L has excellent diagnostic performance for detecting severe wasting and very good performance AUC 0⋅77 (95 % CI 0⋅73, 0⋅81) for detecting moderate wasting. The optimal MUAC/L cut-offs to detect severe wasting <0⋅185 (sensitivity of 91⋅1 % and specificity of 84⋅7 %) and <0⋅201(sensitivity of 73⋅8 % and specificity of 69⋅8 %) for moderate wasting. Thus, MUAC/L can accurately identify wasting in Ethiopian infants with acceptable sensitivity and specificity. As far as we are aware, the present study provides the first MUAC/L cut-off for moderate and severe wasting in infants aged 1–months.

The use of appropriate anthropometric thresholds is based on their association with the ability to predict mortality^(27)^. In the absence of data on mortality, predictive and discriminatory abilities are used to define the anthropometric threshold. Currently, the WHO recommends the adoption of older children's criteria for diagnosing wasting under 6 months^(9)^. Current evidence suggests that MUAC can be used to identify severe wasting among infants aged 1–6 months^(11,12)^. However, a key question challenging the simple use of MUAC-based criteria in infancy is the issues on age and sex independence of the measurement because of the rapid growth speed in younger infants. In children aged 6–59 months, MUAC shows a known bias towards identifying younger and smaller infants as undernourished^(28)^. The height attained at a specific age has its own independent effects on arm measures, regardless of age or sex difference. As a result, arm-for-height reference curves were created for assessing the nutritional status of US children^(29)^.

As of our knowledge, there is a paucity of studies that have assessed the performance of MUAC/L in detecting wasting in infants. In the present study, MUAC/L was considered as an anthropometric parameter to identify wasted (moderately and severely wasted) infants. Using the arm-to-length ratio provides age and sex independent measures because length strongly correlates with age^(30)^. Therefore, MUAC/L can be used to represent age and mid-arm circumference measurements for infants aged under 6 months, where MUAC also correlates with age^(11)^. Similarly, it was demonstrated that MUAC-for-length/height *Z*-score is a better predictor of weight-for-length/height than MUAC based on a fixed cut-off by reporting a reference set of curves for MUAC-for-length/height. According to studies, the QUAC stick technique (adjusting MUAC cut-offs based on height) can be used as a quick method for determining nutrition levels in large populations, as well as a screening tool for malnourished children^(31,32)^. This measurement has numerous practical benefits, particularly in difficult field conditions.

In the present study, MUAC/L cut-offs of <0⋅201 and <0⋅185 were found to be the optimal cut-off points to identify moderate and severe wasting, respectively. The MUAC/L cut-off of <0⋅185 had a sensitivity of 91⋅1 %, a specificity of 84⋅7 % and an AUC of 0⋅87, which indicates that MUAC/L has an acceptable level of accuracy to identify severe wasting. The present study found that MUAC/L has excellent performance (AUC 0⋅92) for detecting severe wasting among infants aged 1–6 months. The Kenyan study showed that MUAC had an AUC of 0⋅75 for identifying infants at risk of in-patient mortality^(33)^. Similarly, the Indian study found that MUAC had a good performance (AUC, 0⋅88) to diagnose severe wasting in infants aged 1–6 months^(22)^. The accuracy of MUAC/L found by the present study is higher compared with the accuracy of MUAC found by our previous study for detecting severe wasting^(12)^.

In another way, the mid-upper arm circumference-to-height ratio was also found to be a reliable and age-independent index with greater applicability as a screening tool for overweight and obese children. These anthropometric measurements had excellent accuracy (ACU ranging from 0⋅920 to 0⋅975), with cut-off values of 0⋅16 (for obesity) and 0⋅145 (for overweight) having high specificity and sensitivity. Another study found that the arm-to-height ratio accuracy levels (ACU) for predicting elevated BMI were over 0⋅85 with an optimal cut-off of 0⋅15, having high sensitivity and specificity.

In the present study, the magnitude of wasting using WLZ was high (34⋅6 %), whereas a previous Indian study showed the magnitude of wasting to be very high (70⋅6 %)^(34)^. The other Indian study also found a very high magnitude of wasting (58⋅2 %) among infants aged 1–6 months^(22)^. The study conducted among Kenyan under 6 months of infants recruited at the time of hospitalization showed a lower magnitude of wasting (22 %) compared with the present study^(11)^. This variation might be due to different geographic settings^(2)^, although MUAC/L identified a similar proportion of wasting among infants aged 1–6 months with WLZ, with a majority overlap between the two indicators. This might help to detect a high proportion of wasted infants in addition to its simplicity relative to WLZ to increase programmatic coverage, particularly in developing countries where the problem is high.

The use of MUAC/L ratio has several advantages in practice for identifying wasting (moderate and severe wasting). First, MUAC/L is not correlated with age, which makes it possible to propose age-independent cut-off points (as we did in the present study), which is easy and feasible to manipulate where there is difficulty in obtaining accurate age. Second, length is simultaneously taken into account in calculating MUAC/L. In the pediatric population, growth is a very important factor for body composition change; therefore, length should always be taken into account. The MUAC/L can prevent misdiagnosis, as the MUAC/L of infants of the same sex, age and arm circumference was lower in long infants than in short infants. Third, MUAC/L measure is not weight dependent – does not require weighing scales, does not require daily-scale calibration and is not sensitive to short-term weight fluctuations. Fourth, MUAC/L is a ratio of two lengths; hence, it is interpretable without unit conversion.

## Strength and limitations

The study's strengths included the use of recommended length and weight measuring equipment to reduce measurement error. Inter- and intra-observer reliability of MUAC, weight and length measurements with their corresponding technical error of measurements (TEM) were computed. The shortcomings of the study are: (1) Some infants were not weighted for length measurement since their length was less than 45 cm. (2) The sample size was calculated with a wasting magnitude of 50 % because there was no published estimate of wasting magnitude in the study setting. (3) During data collection, infants with wasting who were in the community were not taken into account.

## Conclusions

According to present findings, MUAC/L is a highly specific and sensitive measurement for diagnosing severe wasting in infants aged 1–6 months. MUAC/L had excellent diagnostic performance in diagnosing severe wasting and good diagnostic performance in diagnosing moderate wasting. The best MUAC/L cut-offs for identifying moderate and severe wasting are proposed to be 0⋅201 and 0⋅185, respectively. Using MUAC/L could enhance treatment coverage for undernourished Ethiopian infants and infants in other contexts with similar features. We highly advise that present findings be validated before applying the proposed cut-offs in a community setting. Further research utilizing outcomes such as morbidity and mortality risk should be undertaken to assess the accuracy of MUAC/L at the community level.
